# Primary Ewing sarcoma/primitive neuroectodermal tumor of the lung

**DOI:** 10.4322/acr.2020.199

**Published:** 2020-09-02

**Authors:** Devika Gupta, Tathagata Chatterjee, Rohit Tewari, Arti Trehan, Anuradha Ahuja

**Affiliations:** 1 Army Hospital, Department of Laboratory Science & Molecular Medicine, New Delhi, India; 2 Army Hospital, Department of Respiratory Medicine, New Delhi, India

**Keywords:** Ewing sarcoma, extraosseous, Lung

## Abstract

We present the autopsy findings and differential diagnosis in a 42year old male who presented with fever and rapidly progressive respiratory symptoms like breathlessness, nonproductive cough and right sided chest pain. Initial imaging workup done at our hospital revealed a large unilateral tumor with tracheal shift. While being evaluated patient developed facial puffiness, tachypnea suggestive of superior vena cava obstruction. Antemortem biopsy of lung mass was attempted twice and that suggested malignant lesion. Unfortunately, the individual had a rapid downhill course following admission. Post mortem examination was conducted that on opening the thoracic cavity revealed total replacement of right lung tissue by a necrotic growth which was deeply adherent to the rib cage. The contralateral lung as well as all other visceral organs were unremarkable grossly. Histopathology confirmed primary Ewing sarcoma of the lung. We hereby, report a rare case of primary lung Ewing sarcoma diagnosed at autopsy.

## INTRODUCTION

Ewing sarcoma/primitive neuroectodermal tumor(ES/PNET) is a rapidly-progressing tumor grouped under the category of small round blue cell sarcomas.^1^ They occur predominantly in children and adolescence age, with a male predilection.^2^ Common sites of occurrence include long bones of extremities, pelvic bone, chest wall, ribs, and vertebrae.^3^ Rarely extraosseous ES is seen arising in maxilla, kidneys, retroperitoneum, female genital tract and lung.^4^ Primary ES of the lung is very rare, with only about 20 case reports in the literature.^5^

ES/PNET is characterized by the presence of undifferentiated small cell phenotype with a wide variety of morphological differentials that includes rhabdomyosarcoma, monophasic synovial sarcoma, non-Hodgkin lymphoma, neuroblastoma. The diagnosis of the ES/PNET involving primarily the lung is challenging and requires the differential with other pulmonary malignancies sharing similar histological features. Therefore, the diagnosis requires a combination triad of morphology, supported by immunohistochemistry and cytogenetics, as these tumors are characterized by distinct genetic translocation t (11:22) forming EWSR1-ETS fusion chimeric transcription.

Herein, we report the clinicopathological correlation in a 42-year-old man, nonsmoker with no known comorbidities who presented with fever, progressively increasing breathlessness, chest pain, and developed superior vena cava (SVC) syndrome to which he succumbed. On post-mortem examination, he was found to have a massive pulmonary mass, which had almost completely replaced the right lung parenchyma.

### Case Report

**Figure 1:**
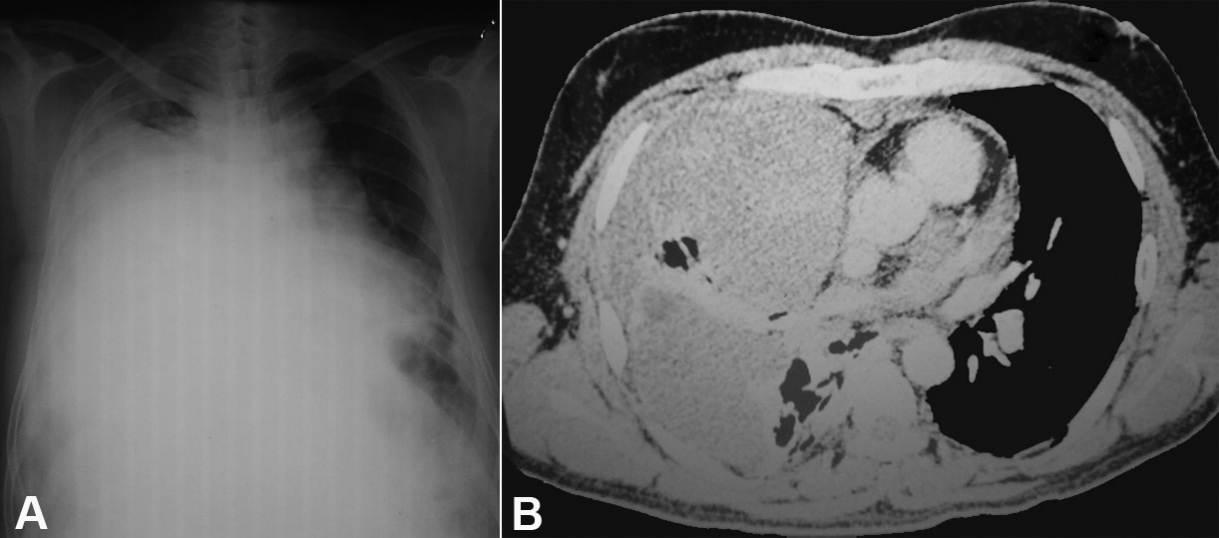
**A –** Chest X-ray showing a homogenous opacity in right hemithorax, and contralateral mediastinal shift; **B –** Thoracic CT showing a pleural based large non-enhancing mass reaching the chest wall anteriorly. Note the preservation of the fat planes. Posteriorly the mass abuts and distorts the right upper lobe bronchus, stretches the ascending branch of right superior pulmonary vein, medially abuts the superior vena cava in its distal course and the ascending aorta at its proximal course and superiorly displaces the horizontal fissure causing the collapse of the middle lobe.

A 42-year-old previously healthy male was referred to our tertiary center complaining of high-grade fever accompanied by chills over the last 20 days. He also complained of progressively increasing breathlessness, nonproductive cough, and right-sided chest pain. There was no history of weight loss or abdominal pain. He denied smoking tobacco. On arrival at our center, he was found to be febrile and dyspneic. His pulse was 120/min, respiratory rate 28/min, and SP02 was 95% at room air. The systemic examination of the chest revealed decreased breath sounds in the right lower zone and coarse crackles in the right infrascapular region. No peripheral or deep chain lymphadenopathy was detected on physical or imaging examinations. The hematological workup showed a leukocyte count of 18,000/µl (reference range [RR]; 4000-12000/μL) with 80% polymorphs. Liver and renal function tests were within normal limits. The serum lactate dehydrogenase (LDH) was 1650 U/L (RR; 150-250U/L). The chest X-ray revealed a heterogeneous opacity in the right pulmonary lower lobe and pleural effusion, which showed to be hemorrhagic after diagnostic thoracocentesis (Figure 1A). The computed tomographic (CT)-guided Tru-cut biopsy attempted in the original-attending hospital showed necrotic tissue. The thoracic CT scan after the second hospitalization revealed a well-defined irregularly, marginated, complex, non-enhancing mass in the right lung, likely pleural based with thickened visceral pleura causing collapse and consolidation of the right lung (Figure 1B). The radiological differentials comprised malignant mesothelioma versus a synovial sarcoma.

**Figure 2:**
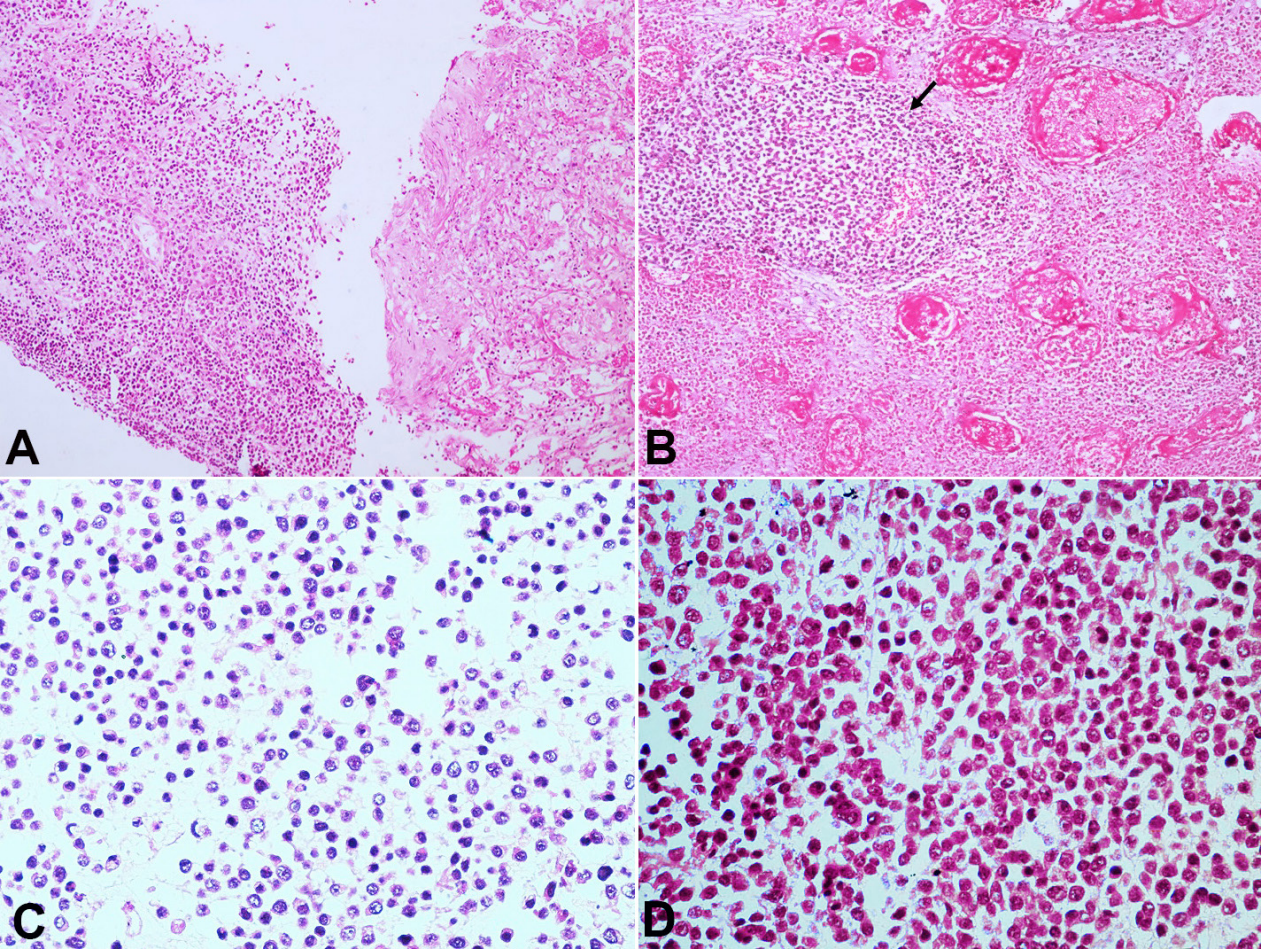
Photomicrographs of: **A –** Antemortem Tru-cut biopsy of the thoracic mass showing proliferating round cells (H&E stain, x200); **B –** Photomicrograph from post mortem lung section shows sheets of small round blue cells having peritheliomatous arrangement with extensive areas of necrosis (H&E stain, x200); **C and D** – the round to oval cells have stippled chromatin, indistinct nucleoli, scant cytoplasm(H&E stain, x200).

A new CT-guided biopsy was attempted and showed atypical cells consistent with an underlying malignant lesion (Figure 2A). The thoracocentesis revealed an exudative and hemorrhagic pleural effusion without malignant cells.

Meanwhile, the patient’s clinical condition deteriorated, he became tachypneic and developed puffiness of the face and altered sensorium and a clinical diagnosis of superior vena cava obstruction was made. He was kept on an antibiotic regimen with Piperacillin-Tazobactam, corticosteroids and controlled ventilation. Bedside 2D echocardiogram failed to evidence a pericardial tamponade, but the mass was seen compressing the right atrium and superior vena cava. On the eighth day of hospitalization, his general condition suddenly worsened, and he expired. Immediately. the autopsy was performed with the working diagnosis of a right lung mass with SVC syndrome, probably due to malignant mesothelioma or a high-grade sarcoma.

### Autopsy Findings

**Figure 3:**
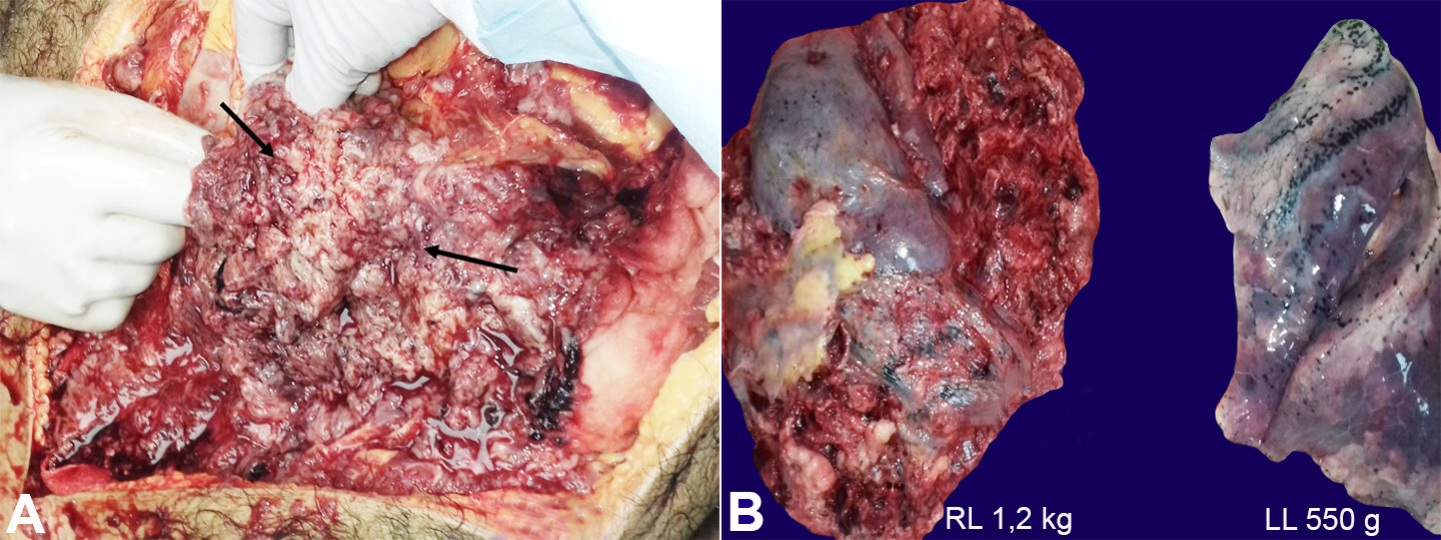
Gross view of **A –** the right lung mass; **B –** in comparison with the left lung, which appears externally normal (RL = right lung; LL = left lung).

External examination revealed proptosis of eyes and midline shift of trachea to the left. On the opening of the chest cavity, the right hemithorax was replaced by a massive hemorrhagic and necrotic tumor mass, which was adherent to the chest wall. In comparison, on the left side, there were 25 ml of straw-colored pleural fluid noted with pleural sheen (Figure 3A). The right lung weighed 1200 g (RR; 360-570 g), and Left lung weighed 550 g (RR; 325-480 g). The right lung was replaced by a large tumor, which was chiefly necrotic, hemorrhagic, and pulpy. Only a portion of the posterior segment of the middle lobe was noted to be intact. The tumor mass pushed the trachea to the left. The left lung was boggy and was oozing frothy blood-stained fluid. The cut section did not show any cavities, abscesses, or tumor deposits (Figure 3B).

The liver was enlarged and weighed 1800 g (RR:1500-1800 g). The external surface was smooth and glistening. No nodularity was noted. The cut surface showed a nutmeg appearance suggestive of passive venous congestion. No mass lesion was identified.

The spleen was enlarged, and weighed 230 g (RR:150-200 g). External and cut surface showed congestion. Both kidneys, pancreas, stomach, intestines, heart and brain were externally and on cut sections unremarkable.

### Microscopic Findings

Histological examination of the lung depicted tumor cells in cohesive sheets, having a peritheliomatous arrangement amidst predominantly necrotic areas. The tumor cells were round to oval, had coarsely stippled chromatin with scant to mildly eosinophilic cytoplasm. Nucleoli were inconspicuous, and mitosis was brisk. Numerous apoptotic bodies were also noted (Figure 22C).

**Figure 4:**
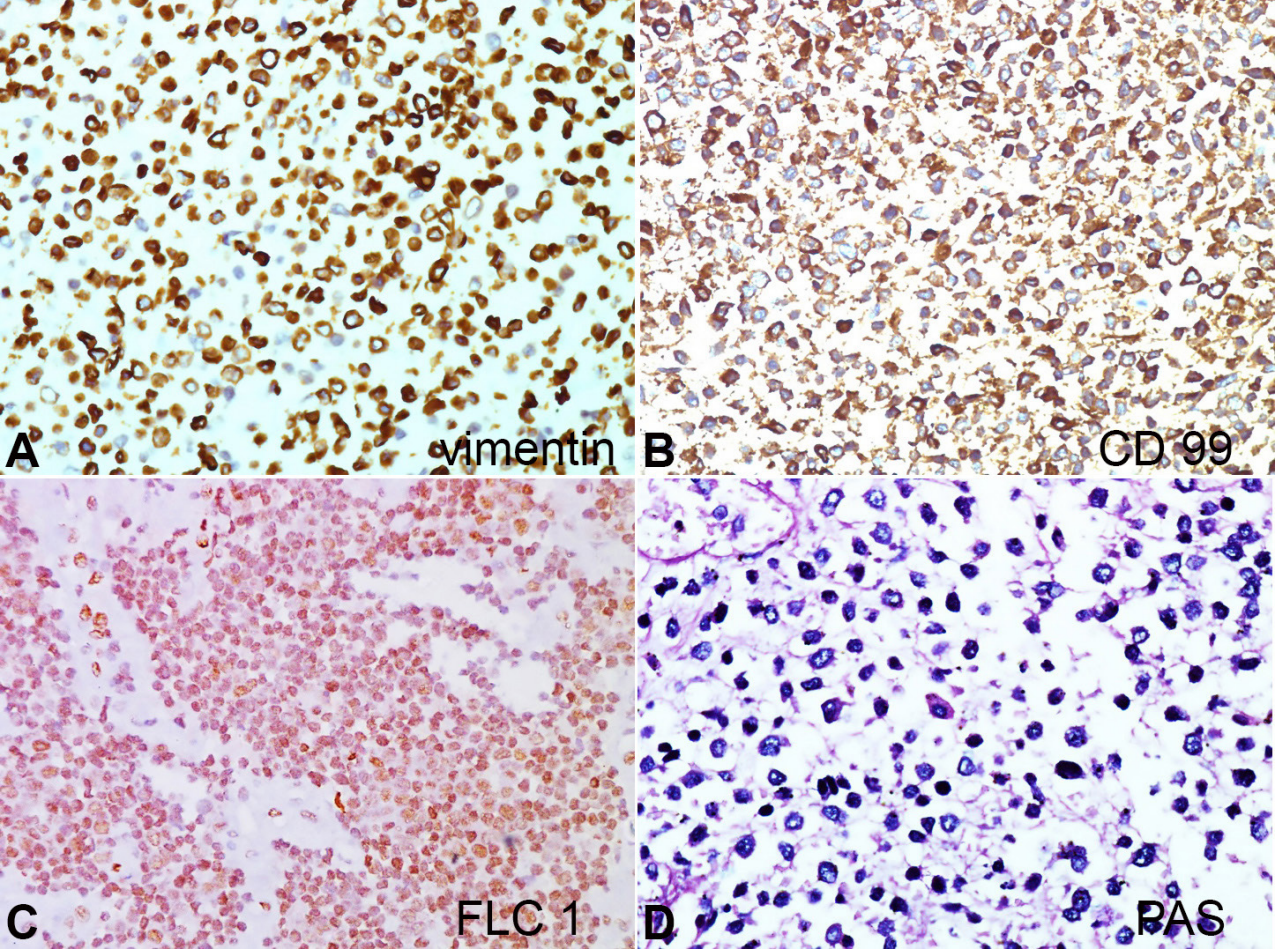
Photomicrograph of the tumor showing the immunohistochemical expression with strong positivity of tumor cells for vimentin (**A**), CD99 (**B**), FLI1 (**C**) and on Periodic Schiff stain (**D**) magenta cytoplasmic uptake. (x400 magnification).

IHC reactions showed the tumor cells to be diffusely positive for Vimentin (strong cytoplasmic positivity), CD99 (strong membranous positivity), and FLI 1(strong nuclear positivity). The tumor cells were diffusely negative for calretinin, WT1, D240, synaptophysin, CD56, TTF1, Tdt, S-100, desmin, myogenin, BCL-2, LCA, and EMA. On Periodic Schiff stain (PAS), the cytoplasm of tumor cells showed strong magenta cytoplasmic positivity (Figure 4AD). Sections from the left upper and lower lobe showed normal air-filled alveoli lined by type II pneumocytes.

No tumor deposits were seen. Sections from the right and left lobe of the liver showed features of passive venous congestion. No tumor deposits were noted. Sections from the spleen showed expansion of red pulp with attenuation of the white pulp. Final diagnosis was primary Ewing sarcoma of right lung causing SVC syndrome.

## DISCUSSION

In 1918, Dr. Arthur Purdy Stout described an undifferentiated tumor arising from the ulnar nerve. He called it PNET because of the presence of rosettes. In 1921 James Ewing, an eminent American Pathologist, reported a tumor affecting long bones, and it was named after him as Ewing sarcoma (ES). Subsequently, these two lesions were described as different entities affecting various sites. However, by 1975, the distinction between ES and PNET began to blur, and it was realized that these two tumors represented two ends of the same histological spectrum and were grouped under the category of Ewing’s family of tumors (EFTs).^6^ It was further substantiated by the fact that they share the same genetic translocation t(11;22)(q24;q12).

The EFT’s are characterized genetically by specific chromosomal translocations resulting in a fusion of the *EWSR1* (22q12) gene with one of the members of the ETS family of transcription factors: the *FLI1* gene (11q24) in 85% of cases and the *ERG* gene (21q22) in 5-10% of cases. Less frequently, *EWSR1* is fused with *FEV* (2q36), *ETV1* (7p21), or *ETV4* (alias *E1AF;* 17q21).^7^ ^,^ ^8^

The ES/PNET arising primarily in the lungs is extremely rare, and a high index of suspicion is required to clinch the early diagnosis and initiate timely treatment. Also, the rarity of primary ES/PNET of lung necessitates a detailed clinical and radiological examination to rule out metastasis from an extrapulmonary site.^9^ Primary Lung ES/PNET was first described by Hammar et al.^10^ in 1989 and a review by Deokar et al.^11^ in 2015, documented only few case reports. To date, all published cases showed unilateral lung involvement, similar to our case. Histologically the ES/PNET cells are small, round with stippled chromatin, indistinct nucleoli, and scant cytoplasm. The morphological differential diagnosis, when the lung is the primary site, includes other small round cell tumors, such as rhabdomyosarcoma, lymphoma, monophasic synovial sarcoma, epithelioid malignant mesothelioma, and small cell lung carcinoma. The demographic profile, morphological features, and IHC pattern of these various lesions are enumerated in Table 1.

**Table 1:** Comparison of Small round cell tumors (SRCTs) arising in Lung

	DEMOGRAPHICS/AGE	MORPHOLOGY	IHC
Ewing Sarcoma	Children/Adolescence Male preponderance	Densely packed small cells with extensive areas of necrosis; interspersed viable cells	Vimentin+ CD99++ FLI1 ++
Rhabdomyosarcoma	Young children Peak in adolescence	Neoplastic cells with varying degrees of resemblance to embryonic skeletal myoblasts	Desmin+ Myogenin+ MyoD+
non-Hodgkin lymphoma	Average age >60yrs	Atypical lymphoid cells with centrocyte, monocytoid, plasmacytoid morphology	LCA+
Synovial sarcoma	Middle aged	Epithelioid monophasic SS comprises of polygonal tumor cells in sheets Necrosis+	PANCK+ EMA+ BCL2+
Malignant mesothelioma	40-60yrs Occupational H/O of asbestos exposure	Aggregates of round to polygonal cells with abundant cytoplasm & fuzzy cell borders	Calretinin+ WT1+ D240+ CK5/6+
Small cell carcinoma lung	>60yrs Smoking+	Small blue cells, prominent nuclear molding Brisk mitosis	CD56+ Syp + CgA+

CgA = Chromogranin; MyoD = Myoblast determination protein; Syp = Synaptophysin; IHC=Immunohistochemistry.

ES cells are positive for vimentin, CD99/MIC 2, and FLI1, as was seen in our case. Though CD99 is highly sensitive for ES, its specificity is low, and can also be seen in rhabdomyosarcoma, synovial sarcoma, and lymphoblastic lymphoma. Antibody against FLI1 is considered to be specific for ES/PNET.^12^

Another differential to be considered, that was lower in our list was malignant melanoma, a known mimicker for various malignant entities. Hence, it is imperative to employ the full IHC panel and negate other tumors before arriving at the definitive opinion. Demonstration of t (11;22) (q24;q12) by fluorescent in situ hybridization and/or reverse transcription-polymerase chain reaction(RT-PCR) is a definitive investigation option.^13^ This investigation was not available in our laboratory.

ES/PNET is an aggressive neoplasm, which is potentially lethal if not intervened timely. The deceased was referred to our hospital at a very advanced stage of the disease and had a rapid downfall in our hospital, intriguingly with a concise time of symptomatic disease. The therapeutic approach requires a combination of surgical resection, followed by chemotherapy and radiotherapy to achieve disease-free survival. Presently newer modalities of targeted immunotherapy against EWS-FLI1 chimeric fusion transcript are being explored.
